# Synthetic Secoisolariciresinol Diglucoside (LGM2605) Protects Human Lung in an Ex Vivo Model of Proton Radiation Damage

**DOI:** 10.3390/ijms18122525

**Published:** 2017-11-25

**Authors:** Anastasia Velalopoulou, Shampa Chatterjee, Ralph A. Pietrofesa, Cynthia Koziol-White, Reynold A. Panettieri, Liyong Lin, Stephen Tuttle, Abigail Berman, Constantinos Koumenis, Melpo Christofidou-Solomidou

**Affiliations:** 1 Division of Pulmonary, Allergy, and Critical Care, Department of Medicine, University of Pennsylvania Perelman School of Medicine, 3450 Hamilton Walk, Stemmler Hall, Office Suite 227, Philadelphia, PA 19104, USA; avelalopoulou@gmail.com (A.V.); ralphp@pennmedicine.upenn.edu (R.A.P.); cjk167@rbhs.rutgers.edu (C.K.-W.); rp856@rbhs.rutgers.edu (R.A.P.); 2 Department of Physiology, University of Pennsylvania Perelman School of Medicine, Philadelphia, PA 19104, USA; shampac@pennmedicine.upenn.edu; 3 Department of Radiation Oncology, University of Pennsylvania Perelman School of Medicine, Philadelphia, PA 19104, USA; Liyong.Lin@uphs.upenn.edu (L.L.); Steve.Tuttle@uphs.upenn.edu (S.T.); abigail.berman@uphs.upenn.edu (A.B.); Costas.Koumenis@uphs.upenn.edu (C.K.)

**Keywords:** antioxidant, cell cycle, human lung sections, inflammation, LGM2605, organ culture, oxidative stress, phase II enzymes, proton radiation, reactive oxygen species, senescence

## Abstract

Radiation therapy for the treatment of thoracic malignancies has improved significantly by directing of the proton beam in higher doses on the targeted tumor while normal tissues around the tumor receive much lower doses. Nevertheless, exposure of normal tissues to protons is known to pose a substantial risk in long-term survivors, as confirmed by our work in space-relevant exposures of murine lungs to proton radiation. Thus, radioprotective strategies are being sought. We established that LGM2605 is a potent protector from radiation-induced lung toxicity and aimed in the current study to extend the initial findings of space-relevant, proton radiation-associated late lung damage in mice by looking at acute changes in human lung. We used an ex vivo model of organ culture where tissue slices of donor living human lung were kept in culture and exposed to proton radiation. We exposed donor human lung precision-cut lung sections (huPCLS), pretreated with LGM2605, to 4 Gy proton radiation and evaluated them 30 min and 24 h later for gene expression changes relevant to inflammation, oxidative stress, and cell cycle arrest, and determined radiation-induced senescence, inflammation, and oxidative tissue damage. We identified an LGM2605-mediated reduction of proton radiation-induced cellular senescence and associated cell cycle changes, an associated proinflammatory phenotype, and associated oxidative tissue damage. This is a first report on the effects of proton radiation and of the radioprotective properties of LGM2605 on human lung.

## 1. Introduction

Lung cancer remains the leading cause of cancer-related death in the USA. Radiation therapy plays a prominent role in the treatment of lung cancer patients, and the standard of care for locally-advanced non-small cell lung cancer (NSCLC) is chemoradiation [1,2]. Innovative advances in radiation therapy have resulted in novel modalities such as proton beam therapy, whereby higher doses of radiation are maintained on the tumor target, and tissue regions beyond the target volume are exposed to relatively low doses [3].

Despite these advances, there is a substantial risk of sub-acute and late side effects from damage to normal tissues, such as the pulmonary conditions of radiation pneumonitis and lung fibrosis, which can cause significant morbidity and mortality. More importantly, proton therapy, despite its focus on target areas of the tumor, has not shown “a survival benefit” for cancer patients due to a significant detrimental effect of radiation on critical organs at risk (OAR), which negates its benefit [4,5]. As of yet no effective intervention or cure for radiation-induced late effects has been developed. 

Over the past decade, we have evaluated radiation effects on the lung and our work has identified dose-dependent distinct lung, pathological, and physiological changes. Specifically, our work in identifying space-radiation-relevant normal tissue damage in lungs exposed to proton radiation revealed significant long term damage. These alterations resemble emphysema, a phenotype of chronic obstructive pulmonary disease (COPD), accompanied by reduced oxygenation induced by proton radiation at 800 days (26 months) following a single exposure to radiation (1 Gy, 2 Gy, and 3 Gy). In this chronic stage of lung injury, we discovered marked imbalances in lung sphingolipid signaling pathways induced by both radiation types, with severely reduced anti-apoptotic and pro-proliferative levels of sphingosine-1 phosphate (S1P), associated with increases in tissue senescence markers. We proposed that the loss of S1P, regulated enzymatically via sphingosine kinases 1 and 2 (SphK1/2) and sphingosine lyase (SphL), is directly linked to maladaptive lung repair and premature/accelerated cellular senescence, and it plays a critical role in radiation-induced lung disease [6]. 

Therefore, this pathway provides the opportunity to develop useful preventive interventions. A major candidate for protection against radiation induced injury as described above is secoisolariciresinol diglucoside (SDG), a biphenolic that scavenges free radicals and triggers a potent endogenous antioxidant response (EAR), preventing radiation-induced acute lung injury [7,8,9,10]. We developed a novel synthetic SDG called LGM2605 [11] that retains the antioxidant properties of the natural compound, but with the advantage of a consistent pharmacodynamic profile [12]. 

We hypothesize that proton radiation induces oxidative stress-mediated lung injury with impaired repair due to maladaptive accelerated senescence, culminating in lung tissue loss and dysfunction; we postulate that lung injury can be prevented by treatment with LGM2605. To test this, we employed human lung tissue in the form of human, precision-cut lung slices (huPCLS), a novel ex vivo human lung organ culture model using tissue from deceased donors [13,14] and investigated the effects of 4 Gy proton radiation exposure of these tissues with and without LGM2605 (50–100 μM) pre-treatment. The radiation dose and LGM2605 concentrations were chosen based on proton therapy protocols (where about 30–60% of the lung receives approximately 4 Gy) and the radioprotective efficacy of LGM2605 (as reported in our earlier studies) [15], respectively. 

This first report of the effects of proton radiation in a model of the human lung establishes that proton radiation-induced inflammation, oxidative stress, and senescence can be significantly diminished by LGM2605. This work lays the groundwork for future studies on radioprotection, as well as mitigation, in response to proton beam exposure in cancer therapy, as well as in proton exposure from solar particle events (SPE) in deep space exploration, whereby crew members are exposed to proton radiation and other harmful galactic cosmic radiation (GCR) [16].

## 2. Results

**Scheme 1:**
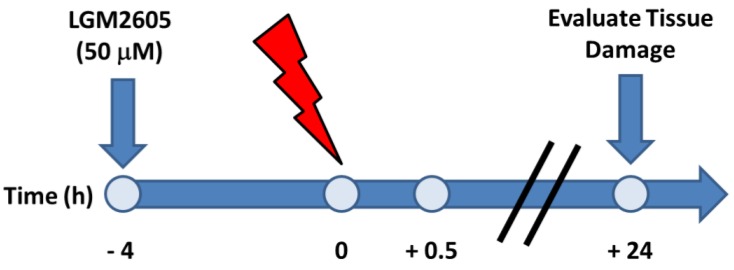
Schematic presentation of the experimental protocol. Human, precision-cut lung sections were exposed to 50 µM synthetic secoisolariciresinol diglucoside (SDG) (LGM2605) 4 h prior to exposure to 4 Gy proton radiation. Lung sections were harvested at 30 min and 24 h post radiation exposure.

We utilized an ex vivo model of organ culture whereby human donor lung slices that are representative of the in vivo tissue were kept alive in culture, as confirmed by the beating cilia of airway epithelial cells lining the bronchioles [17], to study the effect of LGM2605 treatment on proton radiation-induced lung damage, inflammation, and oxidative stress. LGM2605 treatment (50 µM) was initiated 4 h prior to exposure to 4 Gy proton radiation and lung sections were collected at 30 min and 24 h post radiation exposure (Scheme 1).

### 2.1. LGM2605 Prevents the Expression of Proinflammatory Cytokine Gene Levels and Reduces the Induction COX-2 by Proton Radiation in huPCLS

**Figure 1:**
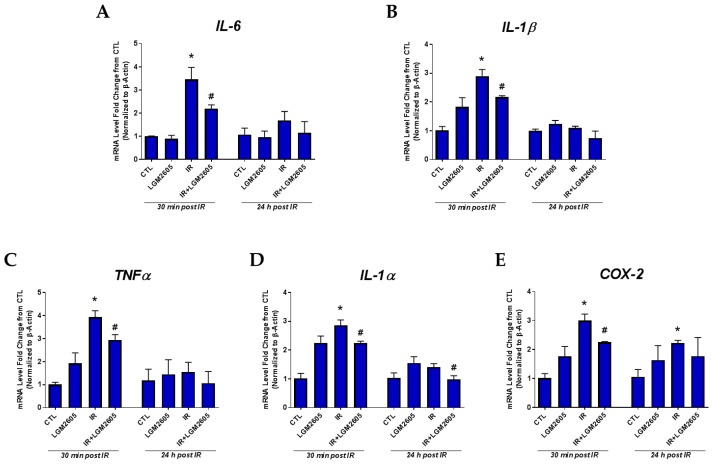
LGM2605 reduces inflammation in proton-irradiated human donor, lung precision-cut lung sections (huPCLS). Human, precision-cut lung sections (*n* = 3–4) from one donor were evaluated at 30 min and 24 h post 4 Gy proton radiation with or without LGM2605 pretreatment for 4 h. Data from one representative donor lung are presented as the average fold change from non-irradiated control ± SEM. Transcript levels of *IL-6* (**A**), *IL-1β* (**B**), *TNFα* (**C**), *IL-1α* (**D**), and *COX-2* (**E**) are normalized to *β-Actin*. * Indicates a statistically significant (*p* < 0.05) difference from the respective non-irradiated control. # Indicates a statistically significant (*p* < 0.05) difference from IR (ionizing radiation-only exposure) (4 Gy proton radiation). IL, interleukin; TNF, tumor necrosis factor; COX, cyclooxygenase; CTL, non-irradiated control.

The acute inflammatory response to proton radiation exposure of lung tissue (huPCLS) was further characterized by determining the mRNA levels of proinflammatory cytokines (*IL-1β*, *IL-6*, *TNFα*, and *IL-1α*) and cyclooxygenase-2 (*COX-2*), an enzyme responsible for the formation of pro-inflammatory moieties [18]. The transcript levels of inflammatory markers, *IL-1β*, *IL-6*, *TNFα*, and *IL-1α*, and *COX-2* in huPCLS were monitored at 30 min and 24 h post 4 Gy proton radiation with or without LGM2605 pretreatment for 4 h and are shown in Figure 1A–E. LGM2605 pretreatment significantly dampened the proton radiation-induced inflammation in huPCLS (36.7% reduction for *IL-6* and 25.1% reduction for *IL-1β*) and significantly (*p* < 0.05) decreased the induction of *COX-2* by 25.0% when evaluated 30 min post radiation exposure.

### 2.2. LGM2605 Boosts Antioxidant Gene Levels by Proton Radiation in huPCLS

**Figure 2:**
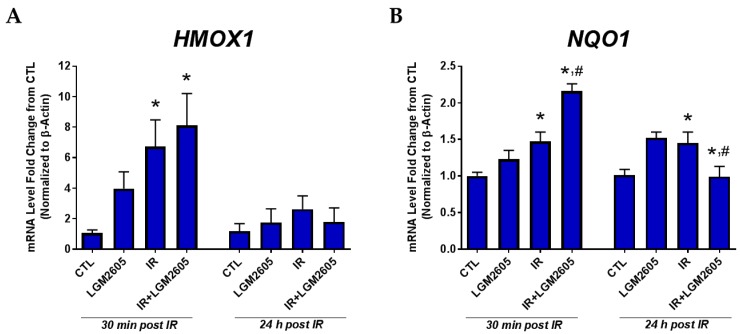
LGM2605 increases expression levels of antioxidant and cell protective genes in proton-irradiated huPCLS. qPCR analysis of *HMOX1* (**A**) and *NQO1* (**B**) was performed on human, precision-cut lung sections (*n* = 3–4) from one donor after 30 min and 24 h post 4 Gy proton radiation with or without LGM2605 pretreatment for 4 h. Data from one representative donor lung are presented as the average fold change from non-irradiated control ± standard error of the mean (SEM). Transcript levels of tested genes are normalized to *β-Actin*. * Indicates a statistically significant (*p* < 0.05) difference from the respective non-irradiated control. # Indicates a statistically significant (*p* < 0.05) difference from IR (4 Gy proton radiation).

Figure 2 depicts the expression of antioxidant genes *HMOX1* and *NQO1* in huPCLS after 30 min and 24 h post 4 Gy proton radiation with or without LGM2605 pretreatment for 4 h. At 30 min post radiation, mRNA levels of the antioxidant gene *NQO1* among proton-exposed huPCLS show a significant (*p* < 0.05) increase over non-irradiated samples (1.47 ± 0.13-fold increase), which was further significantly boosted by the action of LGM2605 (2.16 ± 0.10-fold increase). The effects of both the radiation exposure and the test article were less profound by 24 h post exposure.

### 2.3. LGM2605 Decreases Proton Radiation-Induced Senescence and Biomarkers of Cellular Senescence in huPCLS

**Figure 3:**
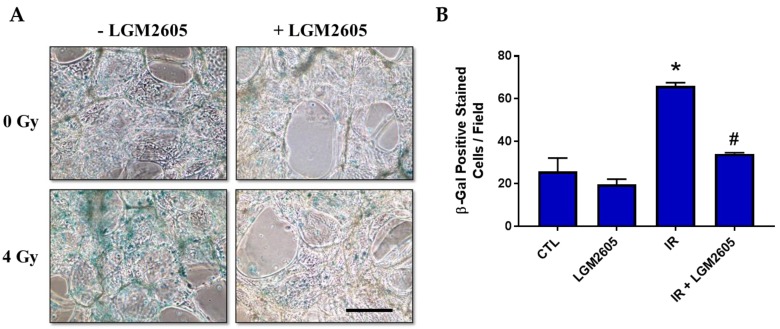
LGM2605 reduces senescence-associated β-galactosidase (SA-β-gal) staining in huPCLS following exposure to 4 Gy proton radiation. huPCLS were exposed to 4 Gy proton radiation and evaluated 24 h later for the detection of senescence-associated β-galactosidase (**A**); 3 random fields per slide were selected and positive-stained cells were counted through the entire thickness of the section by selecting 8 different focus levels. The average number of SA-β-gal positive-stained cells per field are presented as the mean ± SEM (**B**). Scale bar = 150 µm. * Indicates a statistically significant (*p* < 0.05) difference from the respective non-irradiated control. # Indicates a statistically significant (*p* < 0.05) difference from IR (4 Gy proton radiation).

**Figure 4:**
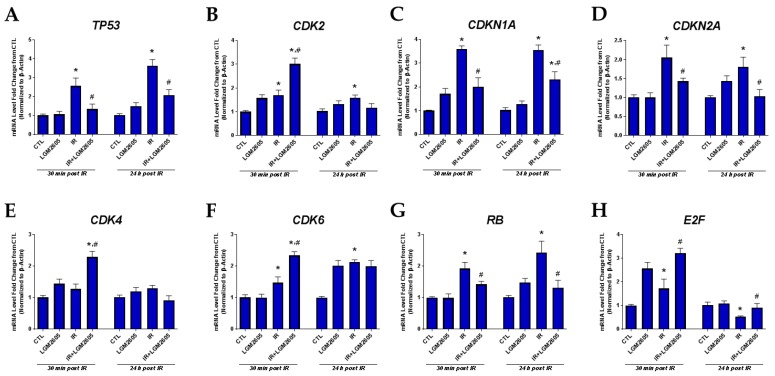
Senescence genes induced by proton radiation are decreased with LGM2605 treatment. qPCR analysis in human, precision-cut lung sections (*n* = 3–4) from one donor after 30 min and 24 h post 4 Gy proton radiation with or without LGM2605 pretreatment for 4 h (**A**–**H**). Data from one representative donor lung are presented as the average fold change from non-irradiated control ± SEM. Transcript levels of tested genes are normalized to *β-Actin*. * Indicates a statistically significant (*p* < 0.05) difference from the respective non-irradiated control. # Indicates a statistically significant (*p* < 0.05) difference from IR (4 Gy proton radiation).

**Figure 5:**
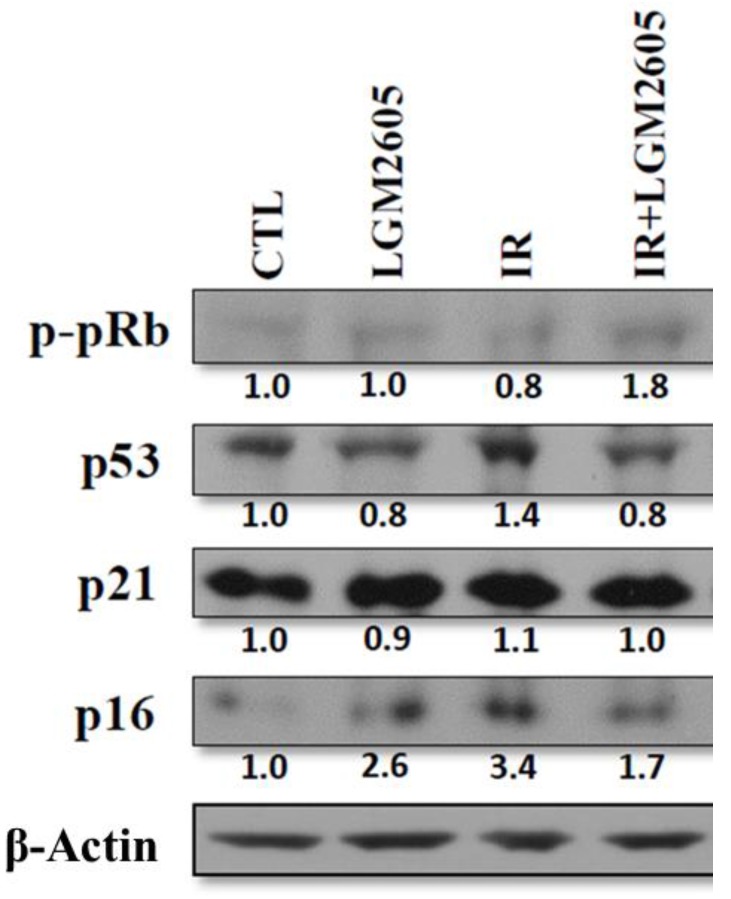
Senescence proteins induced by 4 Gy proton radiation in huPCLS. Western Blot (WB) analysis 30 min post 4 Gy proton radiation exposure of whole tissue lysates from human, precision-cut lung sections (*n* = 3–4) from one donor treated with or without LGM23605 pretreatment. Results shown are from one representative donor lung. Numbers below each WB band designates fold change over control (CTL) after values have been normalized to β-Actin.

Next, we evaluated cellular senescence in huPCLS by staining for senescence-associated β-galactosidase. As shown in Figure 3, exposure to 4 Gy proton radiation significantly (*p* < 0.05) increased senescence-associated β-galactosidase (SA-β-gal) staining (blue staining in Figure 3A), which was significantly reduced by LGM2605 pretreatment to levels comparable to non-irradiated huPCLS. After observing such a robust improvement in proton radiation-induced cellular senescence with LGM2605 pretreatment, we next evaluated molecular biomarkers of cellular senescence both at the message (gene) and the induced protein level. The oncosuppressor gene *TP53* orchestrates the transcriptional response that culminates in senescence and cell death [19]; the protein TP53 that is induced gets stabilized (via post translational modifications) in response to damage/radiation. TP53 can activate cell cycle-arresting genes like cyclin-dependent kinase (*CDKN1A*) [20]. The CDK family of proteins drives the cell cycle by phosphorylating various substrates involved in cell growth and differentiation. CDK4/6 and CDK2 inactivate the retinoblastoma tumor suppressor protein (pRb), which is a “gatekeeper” of the G1-S phase transition. Another member of the CDK family is the *CDKN2A* gene that encodes different transcripts involved mostly in cell cycle regulation and cellular senescence such as the tumor suppressor proteins p16 and p19. Figure 4 and Figure 5 show proton radiation-induced increases in the mRNA and protein levels of TP53, as well as CDKN2A (p16), and that this increase, which is an index of increased senescence, is significantly (*p* < 0.05) abrogated by LGM2605 pretreatment. Additionally, phosphorylation of pRb was boosted by as much as 80% by LGM2605 as compared to untreated irradiated lung slices, in which expression was decreased by 20% from respective non-irradiated controls. This reversal of the hypophosphorylated state of pRb is associated with inhibition of cellular senescence [21]. *CDK2*, *CDK4*, and *CDK6*, which trigger the G1/S phase and thus drive cell proliferation, are significantly (*p* < 0.05) elevated among huPCLS pretreated with LGM2605 and exposed to proton radiation compared to huPCLS, which is exposed to proton radiation alone.

### 2.4. LGM2605 Reduces Proton Radiation-Induced Oxidative Stress in huPCLS

**Figure 6:**
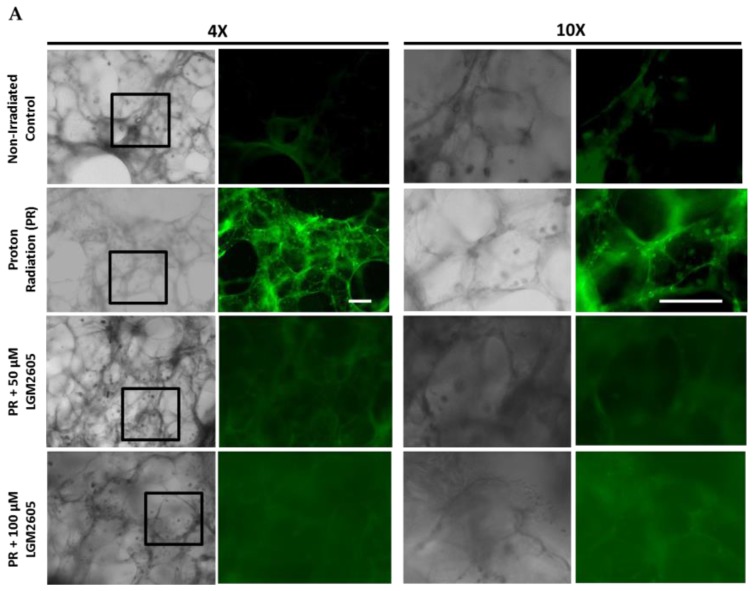

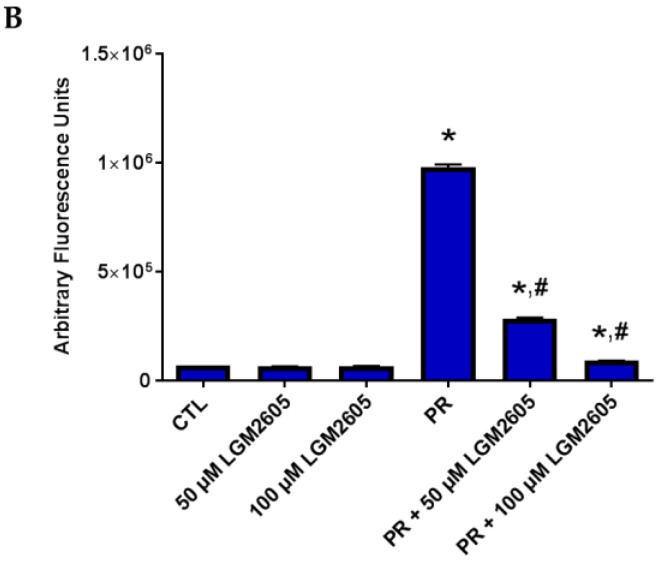
LGM2605 reduces proton radiation-induced oxidative stress in huPCLS. Radiation-induced oxidative stress in huPCLS was monitored by CellROX Green staining and fluorescence. huPCLS were exposed to proton radiation and treated with LGM2605, after which they were labeled with CellROX Green dye (5 μM) for 20 min. This was followed by removal of the dye and resuspension of huPCLS in fresh media and fluorescence imaging of the sections at λ = 488 nm. Images were acquired by 4× and 10× lens. The boxed regions of images acquired at 4× were visualized in 10× to show more structural details. Scale bar = 100 µm (**A**); All images were acquired at the same settings using MetaMorph acquisition software (Version 7.7, Molecular Devices, Downington, PA, USA). The images acquired by 4× lens were used to quantify using MetaMorph and ImageJ software (Fiji Version, National Institutes of Health, Bethesda, MD, USA). The data was acquired from images acquired in 6–7 fields (**B**). Results from one representative donor lung are presented. * Indicates a statistically significant (*p* < 0.05) difference from the respective non-irradiated control. # Indicates a statistically significant (*p* < 0.05) difference from IR (4 Gy proton radiation).

**Figure 7:**
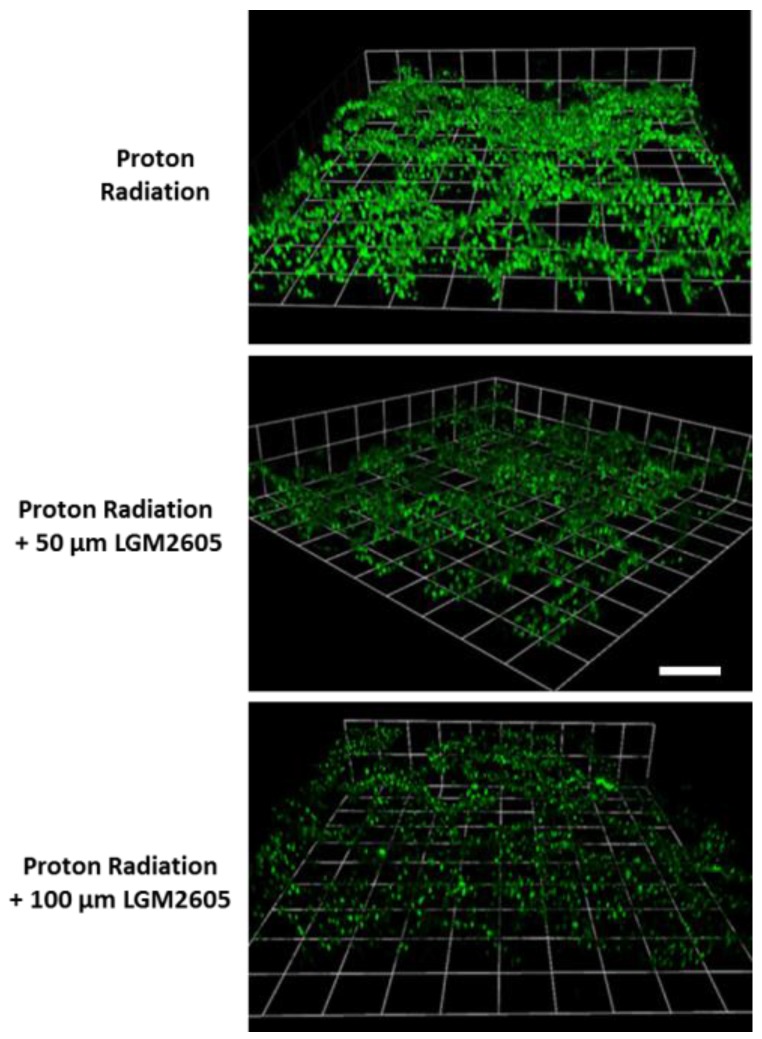
LGM2605 reduces proton radiation-induced oxidative stress in huPCLS. As the huPCLS were ~300 to 350 µm thick, the oxidative stress along the depth of the tissue and its diminution by LGM2605 were also assessed by imaging along the *z*-axis at 10 µm interval throughout the tissue. The *z* stack images acquired on a multiphoton fluorescent microscope were converted into 3D display stacks (using Volocity^®^ Visualization Program, Version 6.3, PerkinElmer, Waltham, MA, USA). Movies of 3D stack were made by ImageJ analysis software. Scale bar = 160 µm. Results from one representative donor lung are presented.

As shown in Figure 6, increase in green fluorescence that arises from the oxidation of the fluorogenic probe that represents cellular oxidative stress implies proton radiation-induced oxidative stress in huPCLS. This cell-permeant dye is non-fluorescent while in a reduced state, and exhibits bright fluorescence in the presence of reactive oxygen species (ROS) or oxidized lipids. LGM2605 at both 50 and 100 µM reduced CellROX Green fluorescence, implying that oxidative stress induced by proton radiation can be significantly reduced by this agent. Figure 7 indicates a 3D image of the irradiated huPCLS, depicting that oxidative damage from proton exposure is full-thickness (i.e., though the entire 300–350 μM lung section) and protection by LGM2605, although added to the medium, i.e., externally, extends through the entire thickness of the exposed tissue.

### 2.5. LGM2605 Reduces a Proinflammatory Phenotype in Proton-Irradiated huPCLS

**Figure 8:**
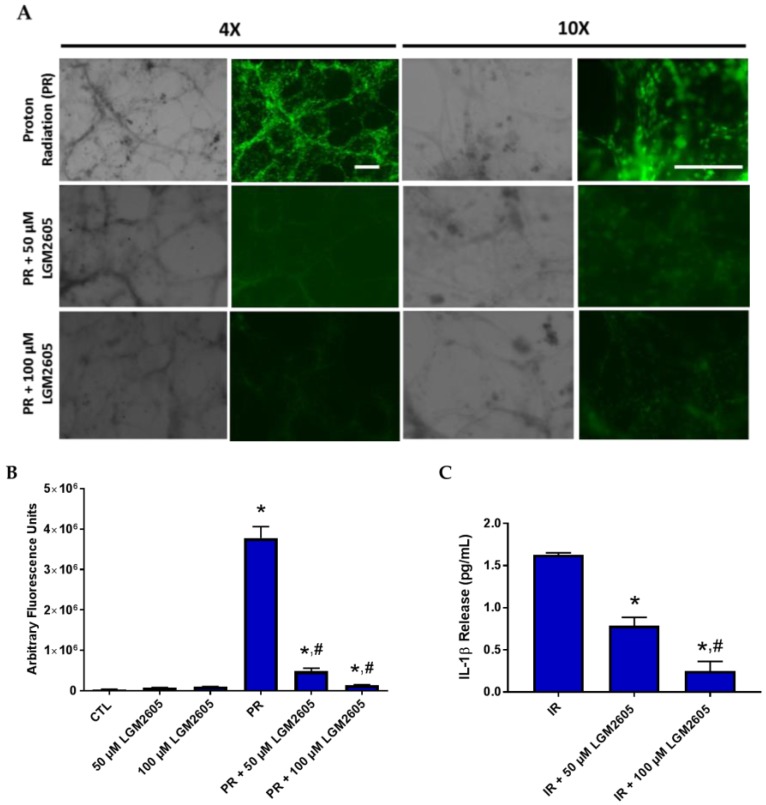
LGM2605 prevents proton-radiation induced inflammation in huPCLS. Radiation-induced inflammation in huPCLS was monitored by intercellular adhesion molecule-1 (ICAM-1) staining and fluorescence. Human, precision-cut lung sections were permeabilized and immunostained with anti-ICAM antibody 24 h post 4 Gy proton radiation exposure. Fluorescence imaging of the sections at λ = 488 nm using 4× and 10× lens was carried out. Scale bar = 100 µm (**A**); All images were acquired at the same settings using MetaMorph acquisition software. The images acquired by 4× lens were used to quantify using MetaMorph and ImageJ software. The data was acquired from images acquired in 6–7 fields (**B**); IL-1β release, as determined by enzyme-linked immunosorbent assay (ELISA), was determined at 24 h post radiation (**C**). Results from one representative donor lung are presented. * Indicates a statistically significant (*p* < 0.05) difference from the respective non-irradiated control. # Indicates a statistically significant (*p* < 0.05) difference from IR (4 Gy proton radiation).

Having observed an increase in the mRNA levels of proinflammatory cytokines, such as *IL-1β* and *IL-6*, following 4 Gy proton radiation exposure, we proceeded to further investigate the proinflammatory phenotype induced by proton radiation by evaluating the expression of intercellular adhesion molecule-1 (ICAM-1) in huPCLS following radiation. As shown in Figure 8, ICAM-1 expression is increased in huPCLS at 24 h post radiation exposure, noted by the increase in green fluorescence (Figure 8A). LGM2605 pretreatment led to a dose-dependent decrease of proton radiation-induced ICAM-1 expression (50 µM LGM2605 pretreatment led to an 87% reduction in proton radiation-induced ICAM-1 expression and 100 µM LGM2605 pretreatment led to a 96% reduction in proton radiation-induced ICAM-1 expression) (Figure 8B).

The observed decrease in ICAM-1-induced expression following proton radiation exposure prompted us to look at the release of a key inflammatory cytokine, IL-1β, by proton-irradiated huPCLS. As shown in Figure 8C, LGM2605 pretreatment significantly (*p* < 0.05) reduced IL-1β release by 52% (50 µM LGM2605) and 85% (100 µM LGM2605).

### 2.6. LGM2605 Does Not Impair Tumor Cell Eradication by Proton Radiation

Exposure of cancer cells to ionizing radiation induces cellular senescence, thus inhibiting tumor growth and progression [22]. Specifically, modulating radiation-induced senescence of non-small cell lung cancer cells has been investigated as a mechanism of overcoming radioresistance [23,24]. As a confirmation that LGM2605 pretreatment does not impair the tumoricidal effect of proton radiation exposure, A549 human lung adenocarcinoma cells were exposed to 4 Gy proton radiation and evaluated for radiation-induced cellular senescence by staining for senescence-associated β-galactosidase.

**Figure 9:**
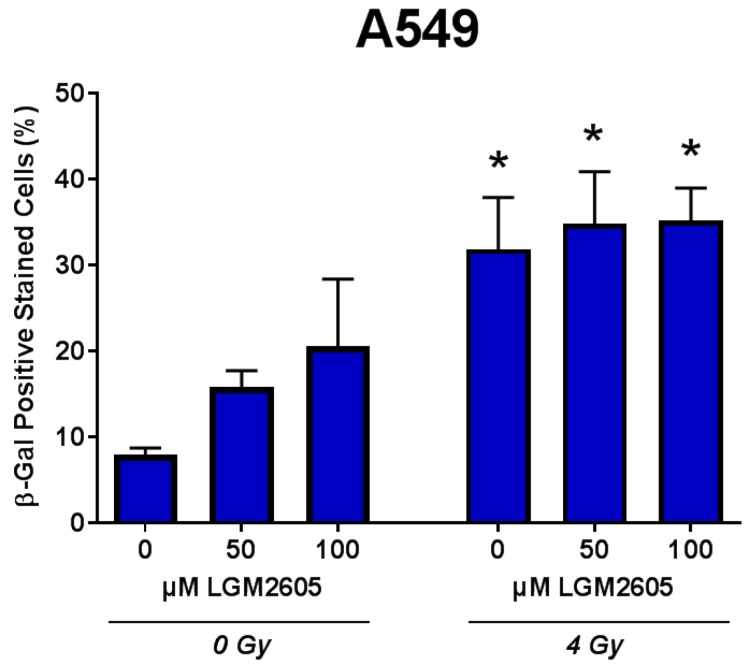
LGM2605 does not impair proton radiation-induced cellular senescence in A549 human lung adenocarcinoma cells. 4 h prior to 4 Gy proton radiation exposure, A549 cells were treated with 0 µM, 50 µM, or 100 µM LGM2605. 24 h following radiation exposure, the cells were stained for senescence-associated β-galactosidase. The blue-stained senescent cells were viewed by light microscopy and positive-stained cells were counted from 5 randomly selected fields. The average number of β-galactosidase positive-stained cells per field are presented as the mean ± SEM. * Indicates a statistically significant (*p* < 0.05) difference from the respective non-irradiated control.

As shown in Figure 9, treatment with LGM2605-alone induced a dose-dependent increase in cellular senescence in A549 cells without radiation proton radiation exposure. Regardless of the dose of LGM2605, proton radiation induced a significant (*p* < 0.05) increase in the percentage of SA-β-gal positive-stained cells (31.8 ± 6.0% with 0 µM LGM2605, 34.8 ± 6.0% with 50 µM LGM2605, and 35.2 ± 3.8% with 100 µM LGM2605). No significant differences between A549 irradiated cells treated with different doses of LGM2605 were observed. These findings are supported by our previous work, where we showed how the radioprotective properties of the lignan SDG did not prevent eradiation of lung tumors from radiation exposure [25].

## 3. Discussion

**Figure 10:**
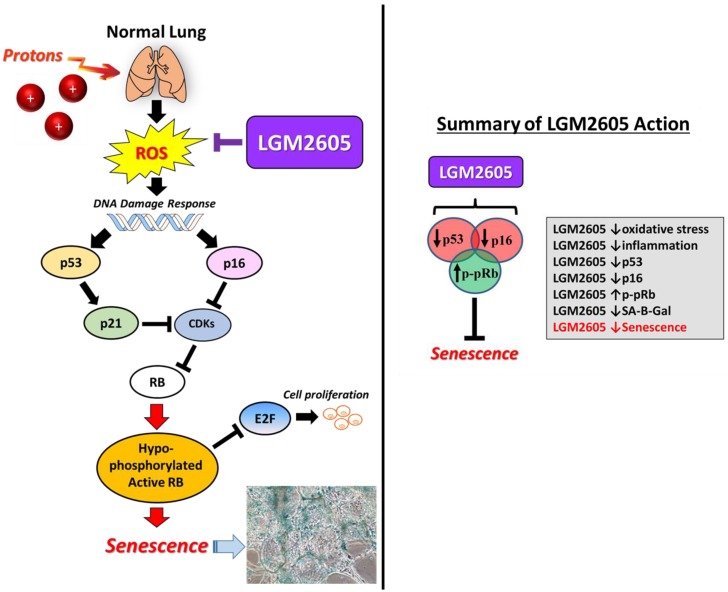
Schematic representation of LGM2605’s blockade of senescence markers induced by proton radiation of normal lung tissue/cell. Arrow-headed lines indicate activation and bar-headed lines indicate inhibition. ROS, Reactive oxygen species; CDK, cyclin-dependent kinase; RB, retinoblastoma; E2F, E2 factor.

Unwanted exposure to radiation of normal tissues, and especially of radiosensitive organs such as the lung, can occur during radiotherapy to treat malignancies, in radiological accidents, and in terroristic acts involving radioactive material, or during space travel where crewmembers may be exposed to galactic cosmic radiation (GCR) and protons from solar particle events (SPEs). Radiation exposure is an identified potential health risk to crewmembers during space travel; however, its effects on the pulmonary system are largely unexplored, constituting a noticeable knowledge gap. Specifically, unrecognized GCR-induced lung damage could lead to chronic breathing problems in crewmembers, such as cough, dyspnea, and decreased exercise capacity [26]. Using a mouse model of GCR and proton radiation exposure, our group identified dose-dependent, distinct, lung pathological and physiological changes resembling chronic obstructive pulmonary disease (COPD) emphysema, accompanied by reduced oxygenation, induced by all GCR and proton radiation types tested, and evaluated more than two years following a single exposure to radiation [6]. The current study aimed to extend those findings using a novel human lung model whereby ultrathin slices (<200 µm) from human donor lungs are kept alive in an ex vivo organ culture system for up to 7 days from procurement. This allowed us to test acute changes to lung tissues following exposure to radiation and to elucidate mechanisms of tissue damage inked to delayed effects such as the ones observed earlier [6]. Importantly, this study evaluated the properties of a novel synthetic agent found to be radioprotective by ROS scavenging, nuclear factor (erythroid-derived 2)-like 2 (Nrf2) pathway activation, and endogenous antioxidant defense boosting, as discovered more recently by scavenging of radiation-induced active chlorine species. We show here for the first time that LGM2605 is an effective radioprotecting agent in human lung tissues exposed to proton radiation. Additionally, we identified the antioxidant and anti-senescence action of this agent, a mechanism that could translate to downstream tissue protection from late effects of radiation (Figure 10). This work provides the basis for such work to be undertaken in future studies.

It has long been known that exposure of normal tissues surrounding the tumor regions to radiation is an unwanted side effect of radiotherapy, which is a major treatment option in lung cancer management [27]. To prevent these complications in the form of fibrosis and the spread of secondary or metastatic cancers, novel innovative radiotherapies such as proton therapy have become an area of growing research. Proton therapy involves less scatter of protons as compared to X-ray/γ radiation. The energy and acceleration of protons can be controlled precisely if maximum (tumoricidal) energy is delivered on the cancer tissue. Despite better control on radiation, proton therapy does not necessarily translate into a survival benefit for the cancer patient due to a significant detrimental effect of radiation on critical organs such as the lung [28,29]. Additionally, radiation-induced complications have very long latency times. For instance, the development of cardiovascular complications after radiotherapy for breast cancer irradiation generally takes between 5–20 years. Therefore, interventions pre- and post-radiation that can regulate proton radiation-induced signaling in normal tissue are being sought. Findings from the current study could potentially benefit cancer patients undergoing radiotherapy as pre-treatment with LGM2605 may prove radioprotective from unwanted side effects. 

Indeed, the lignan SDG was shown in rodent models of lung damage to be a potent protector from radiation-induced lung toxicity when given prior to radiation exposure [30], and even post-radiation exposure [31]. SDG, now synthesized as LGM2605 to ensure constant pharmacodynamics, can be employed as a protective agent in proton therapy. The huPCLS preparation used in this study emulates and preserves essential characteristics of human lung; thus, we reasoned that it would be a valuable tool used to detect proton radiation-induced damage to the human lung. In this study, we monitored inflammation, oxidative stress (and antioxidant status), and senescent markers post protein beam exposure. Radiation-induced senescence in non-malignant, normal cells such as non-proliferating endothelial cells has been known and is mediated in part by ROS generation and p53 activation [32,33]. Of note, exposure of normal tissues and cells to moderate levels of radiation (<10 Gy), as encountered in normal tissues outside the targeted tumor tissue in radiotherapy patients or during space travel, induces senescence via multiple pathways [33]. Berman and coworkers [34] have reported an in-silico comparative analysis of passive scattering proton therapy (PSPT) and intensity modulated proton therapy (IMPT) with intensity-modulated photon beam radiotherapy (IMRT) radiation treatment. The study reported that 30–60% of the lung receives ~400 cGy and was thus selected as a relevant dose to study lung responses to proton exposures.

## 4. Materials and Methods

### 4.1. Human, Precision-Cut Lung Slices (huPCLS)

huPCLS were prepared as previously described [13,14]. Briefly, whole human lungs from non-asthma donors were obtained from the National Disease Research Interchange (Philadelphia, PA, USA) and were dissected and inflated using 2% (*w*·*v*^−1^) low melting point agarose. Once the agarose set, the lobe was sectioned, and cores of 8 mm diameter were made. The cores that contained a small airway by visual inspection were sliced at a thickness of 350 μm (Precisionary Instruments VF300 Vibratome, Greenville, NC, USA) and collected in wells containing supplemented Ham’s F-12 medium. The cores generated were randomized as to the location in the lungs they were derived from, so the slices generated came from throughout the lungs and not one specific area. Slices containing contiguous segments of the same airway served as controls and were incubated at 37 °C in a humidified air:CO_2_ (ratio of 95:5) incubator. Sections were rinsed with fresh media 2–3 times on days 1 and 2 to remove agarose and endogenous substances released that may confound the production of inflammatory mediators [13].

### 4.2. Irradiation Procedure

huPCLS were irradiated with a spread out proton Bragg peak (SOBP) in the Robert’s Proton Therapy facility at the University of Pennsylvania with a 20 cm × 20 cm beam area encompassing a 3 × 3 square array of 60 mm dishes with huPCLS. Delivery modality was a scanned pencil beam, with dose delivered in sequentially shallower energy layers to generate an SOBP. Beam energy and SOBP width are chosen to produce a dose-averaged LET at the cell irradiation depth of 3–5 keV/µm for a total dose 4 Gy. The irradiation depth was determined with proton range-verified water equivalent thickness. The proton beam output is calibrated according to the International Atomic Energy Agency code of practice for absorbed dose to water [35] using ionization chambers with traceable calibration certificates from the National Institute of Standards and Technology (NIST).

### 4.3. LGM2605 Treatment

LGM2605 (50 and 100 μM) was added to the huPCLS 4 h prior to proton radiation. The schema for LGM2605 treatment, as well as for the measuring of the various indices of inflammation and oxidative damage, are shown in Scheme 1. 

### 4.4. Western Blots

Total protein content from huPCLS was isolated using Trizol by following the manufacturer’s suggested protocol for sequential precipitation of RNA, DNA, and proteins (Invitrogen, Carlsbad, CA, USA). Briefly, after homogenization of the lung sections and incubation with chloroform, samples were allowed to form three different phases from which the lower organic phase was isolated. Protein content was precipitated using isopropanol, washed to remove impurities, and resuspended and quantified using BCA Protein Assay (Thermo Scientific, Waltham, MA, USA). Samples were loaded on 8–12% NuPAGE gel (Invitrogen, Carlsbad, CA, USA) and proteins were transferred on PolyScreen PV transfer membrane (PerkinElmer Life Sciences, Boston, MA, USA). After membrane blocking with 5% BSA, protein levels of human senescent proteins p21 Waf1/Cip1 (product number 2948, Cell Signaling Technology, Danvers, MA, USA), p16 INK4A (product number 4824, Cell Signaling Technology), p53 (product number 9282, Cell Signaling Technology), and Phospho-Rb (product number 9307, Cell Signaling Technology) were detected using rabbit and mouse monoclonal antibodies, following manufacturer recommended dilutions. Peroxidase-conjugated donkey anti-rabbit IgG (code 711-035-152, Jackson ImmunoResearch Laboratories, Inc., West Grove, PA, USA) and goat anti-mouse IgG (code 115-035-003, Jackson ImmunoResearch Laboratories, Inc.) were used as secondary antibodies. Bands were visualized using Western Lighting Chemiluminescence Reagent Plus (PerkinElmer Life Sciences) and quantified with ImageJ software (Fiji Version, National Institutes of Health, Bethesda, MD, USA). Tissue preparation from the limited lung slices available from each donor lung resulted in restricting protein amounts, which only allow a single western blot with no possibility of repeat.

### 4.5. Oxidative Stress

Monitoring of huPCLS for oxidative stress was carried out by fluorescence using CellROX Green (Thermofisher Scientific, Eugene, OR, USA). After proton exposure, huPCLS were labeled with 5 μM CellROX Green dye and incubated for about 20 min, after which these were washed with RPMI buffer. HuPCLS were placed on clear glass coverslips and imaged using a Nikon fluorescence microscope (Nikon Diaphot TMD, Melville, NY, USA). For each condition, the data was quantified over 3–4 fields using MetaMorph acquisition software (Version 7.7, Molecular Devices, Downington, PA, USA). Images were acquired at excitation of 488 nm. All images were acquired at the same settings and the CellROX Green fluorescence quantified by integrating the intensity of fluorescence across the entire fields.

### 4.6. Images in Z-Stack

As the huPCLS were ~300–350 μm thick, the oxidative stress along the depth of the tissue and its diminution by LGM2605 were also assessed by imaging along the *z*-axis at 10 μm micron interval throughout the tissue. Images were acquired at excitation of 488 nm to record the CellROX Green fluorescence. The imaging data was presented as a 3D image, as well as in the form of a video file at fifteen frames, single *z* plane acquisition. All *z* stack images were acquired using a Leica STED 3X Super-Resolution Microscope equipped with a LAS X software version 3.1 (Wetzlar, Germany) for image acquisition and Huygens Professional software (Version 4.5, Scientific Volume Imaging, Hilversum, The Netherlands) for deconvolution. Images were converted into single stacks using ImageJ analysis software (NIH). This software was also used to make movies, whereby the images were rotated along the *xy* axes. Finally, 3D display stacks were made by fitting in the ImageJ stacks into Volocity^®^ Visualization Program (Version 6.3, PerkinElmer, Waltham, MA, USA). Scale bar = 160 µm. 

### 4.7. RNA Isolation and Gene Expression Analysis

Total RNA was isolated from huPCLS and qPCR analysis was performed as previously described [15,25,36] using individual TaqMan^®^ Probe-Based Gene Expression Assays (Applied Biosystems, Life Technologies, Carlsbad, CA, USA) selected for proinflammatory cytokines (*IL-1β*, *IL-6*, *TNFα*, and *IL-1α*), inflammation (*COX-2*), relevant cytoprotective and antioxidant enzymes (heme oxygenase-1 (*HMOX1*) and NADPH:quinone oxidoreductase-1 (*NQO1*), and cell cycle markers (*TP53*, *CDK2*, *CDKN1A*, *CDKN2A*, *CDK4*, *CDK6*, *RB*, and *E2F*). Transcript levels of tested genes were normalized to *β-Actin*.

### 4.8. Senescence-Associated β-Galactosidase Staining

Proton radiation-induced cellular senescence of A549 human lung adenocarcinoma cells was evaluated by senescence-associated β-galactosidase (SA-β-gal) staining. Briefly, 30,000 cells were seeded in 60 mm dishes and pretreated with either 0, 50, or 100 µM LGM2605 4 h prior to 4 Gy proton radiation. At 24 h post radiation, cells were fixed and stained using the Senescence β-Galactosidase Staining Kit (Cell Signaling Technology) according to the manufacturer’s instructions. The blue-stained senescent cells were viewed by light microscopy and positive-stained cells were counted from 5 randomly selected fields. SA-β-gal-stained huPCLS were similarly evaluated by light microscopy. Eight focus levels in 3 randomly-selected fields were evaluated per slide under 400× magnification.

### 4.9. Intercellular Adhesion Molecule (ICAM) Quantification

Human, precision-cut lung sections were permeabilized and immunostained with anti-ICAM antibody (1:250) 24 h post irradiation with 4 Gy protons, huPCLS. Secondary antibody (goat anti mouse) with FITC label was used. Fluorescence imaging of the sections at λ = 488 nm using 4× and 10× lens was carried out. All images were acquired at the same settings using MetaMorph acquisition software. The images acquired by 4× lens were used to quantify using MetaMorph and ImageJ software. The data was acquired from images acquired in 6–7 fields.

### 4.10. IL-1β Cytokine Release

Levels of proinflammatory cytokine, IL-1β, were determined in tissue culture medium at 24 h post 4 Gy proton radiation exposure using an enzyme-linked immunosorbent assay (ELISA). Samples were run undiluted in triplicate, and the assay was performed according to manufacturer's instructions (BD biosciences, San Jose, CA, USA). Levels of IL-1β are reported as picograms per milliliter (pg/mL) of culture medium.

### 4.11. Statistical Analysis

All results shown are from multiple lung sections sampled from different sites, yet from the same core biopsy (*n* = 3–4 sections), and are reported as the mean ± the standard error of the mean (SEM). Of note, due to large inter-donor variability, 5 different donor lungs were evaluated for their sensitivity to radiation exposure using induction (>2-fold change over non-irradiated control) of senescence-relevant genes using qPCR at both 30 min and 24 h as a measure of selection, and just two IR-responder lungs were selected to be evaluated further with WB. The selected donor lungs were then evaluated for the protective effects of the test agent (LGM2605). Data are normally distributed and statistically significant differences were determined by one-way analysis of variance (ANOVA), followed by Tukey’s multiple comparisons tests using GraphPad Prism version 6.00 for Windows, GraphPad Software, La Jolla, CA, USA, www.graphpad.com. All pairwise comparisons were performed within each respective time point post radiation exposure, and statistically significant differences are reported as: * indicates a statistically significant (*p* < 0.05) difference from the respective non-irradiated control, and # indicates a statistically significant (*p* < 0.05) difference from IR (4 Gy proton radiation).

Gene expression data are reported as the fold change from CTL (no LGM2605 and no exposure to proton radiation) for each respective time point. Statistically significant differences in mRNA levels were determined using nonparametric tests (Mann–Whitney tests) due to the non-normality of the fold change data. Statistically significant differences were determined at α = 0.05.

## 5. Conclusions

In summary, our findings provide evidence that LGM2605 treatment significantly reduces proton radiation-induced oxidative lung damage and cellular senescence, while ameliorating an overall proinflammatory phenotype in a human lung organ culture model system. Additionally, LGM2605 pretreatment of proton-irradiated human lung slices significantly upregulates antioxidant genes and downregulates proinflammatory cytokine gene levels. LGM2605 protects huPCLS from a senescent-like phenotype induced by proton radiation. The senescent-like phenotype regulated at the gene and protein level by p53, members of the CDK family, p21, and p16 is significantly reduced by LGM2605 pre-treatment. Additionally, radiation-induced cellular senescence has been associated with radiation-induced late effects. We, therefore, speculate that abrogation of senescence acutely, as shown by the current study, may have downstream protective effects. LGM2605 may be a candidate countermeasure agent for space-relevant radiation exposures, as well as in adverse effects of radiotherapy for cancer eradication.

## Abbreviations

CDK	cyclin-dependent kinase
COPD	chronic obstructive pulmonary disease
COX-2	cyclooxygenase-2
EAR	endogenous antioxidant response
ELISA	enzyme-linked immunosorbent assay
GCR	galactic cosmic radiation
HMOX1	heme oxygenase-1
huPCLS	human, precision-cut lung slices
ICAM	intercellular adhesion molecule
IL-1α	interleukin-1α
IL-1β	interleukin-1β
IL-6	interleukin-6
IMPT	intensity modulated proton therapy
IMRT	intensity modulated photon beam radiotherapy
LGM2605	synthetic SDG
Nrf2	nuclear factor (erythroid-derived 2)-like 2
NQO1	NADPH: quinone oxidoreductase-1
OAR	organs at risk
PBS	phosphate-buffered saline
qPCR	quantitative polymerase chain reaction
RNS	reactive nitrogen species
ROS	reactive oxygen species
SA-β-gal	senescence-associated β-galactosidase
S1P	sphingosine-1 phosphate
SDG	secoisolariciresinol diglucoside
SphK1	sphingosine kinase 1
SphK2	sphingosine kinase 2
SpHL	sphingosine lyase
TNFα	tumor necrosis factor α
